# Photometric stereo data for the validation of a structural health monitoring test rig

**DOI:** 10.1016/j.dib.2024.110164

**Published:** 2024-02-06

**Authors:** Jennifer Blair, Bruce Stephen, Blair Brown, Stephen McArthur, David Gorman, Alistair Forbes, Claire Pottier, Jack McAlorum, Hamish Dow, Marcus Perry

**Affiliations:** a Institute for Energy and Environment, Department of Electronic and Electrical Engineering, University of Strathclyde, Glasgow, G1 1XJ, UK; b National Physical Laboratory, Teddington, TW11 0LW, UK; c Department of Civil and Environmental Engineering, University of Strathclyde, Glasgow, G1 1XJ, UK

**Keywords:** Experiment virtualisation, Intersystem comparison, Coordinate measurement machine, Non-destructive testing, Uncertainty quantification, Civil engineering

## Abstract

Photometric stereo uses images of objects illuminated from various directions to calculate surface normals which can be used to generate 3D meshes of the object. Such meshes can be used by engineers to estimate damage of a concrete surface, or track damage progression over time to inform maintenance decisions. This dataset [1] was collected to quantify the uncertainty in a photometric stereo test rig through both the comparison with a well characterised method (coordinate measurement machine) and experiment virtualisation. Data was collected for 9 real objects using both the test rig and the coordinate measurement machine. These objects range from clay statues to damaged concrete slabs. Furthermore, synthetic data for 12 objects was created via virtual renders generated using Blender (3D software) [2]. The two methods of data generation allowed the decoupling of the physical rig (used to light and photograph objects) and the photometric stereo algorithm (used to convert images and lighting information into 3D meshes). This data can allow users to: test their own photometric stereo algorithms, with specialised data created for structural health monitoring applications; provide an industrially relevant case study to develop and test uncertainty quantification methods on test rigs for structural health monitoring of concrete; or develop data processing methodologies for the alignment of scaled, translated, and rotated data.

Specifications Table

**Table:** 

Subject	Civil and Structural Engineering
Specific subject area	Structural health monitoring through application of photometric stereo
Data format	.jpg (images from test rig camera and renders) .png (images of normal and albedo generated by photometric stereo code) .txt (meta data from test rig and virtual rig) .csv (coordinate data for mesh vertices) .blend (Blender file containing virtual rig and meshes) .ply (meshes created by photometric stereo code) .stl (meshes created by PolyWorks software) .npy (normal vector data from photometric stereo code)
Type of data	Image (pictures taken of objects by the test rig, rendered images from the virtual experiment in Blender, images of the object normal and albedo generated by the photometric stereo code) Text (Meta data concerning the virtual and real test rig set up) Numerical (Coordinate data for all vertices in the 3D meshes) 3D meshes (Meshes generated by the photometric stereo code, PolyWorks software, the virtual rig and virtual objects)
Data collection	Data of physical objects was collected by the photometric stereo test rig which consists of LED lighting and a camera (Blackfly USB bfs-u3-200s6c, 8 mm lens), and python code; and a coordinate measurement machine and PolyWorks software (Hexagon ‘Absolute Arm 7-Axis with Absolute Scanner AS1’). The virtual rig was created in Blender (version 3.4) where renders were created of virtual objects.
Data source location	Data of the physical objects were scanned using a coordinate measurement machine at National Physical Laboratory in Huddersfield, UK. The same objects were scanned by the photometric stereo test rig at the University of Strathclyde, UK.
Data accessibility	Repository name: PURE DOI: 10.15129/b571ee22-1d37-484d-b2fb-5496e5d4315e Direct URL to data: https://doi.org/10.15129/b571ee22-1d37-484d-b2fb-5496e5d4315e Instructions for accessing these data: Data is publicly available from the provided links

## 1 Value of the Data

• This dataset provides a set of benchmarks for understanding the uncertainties between 3D geometries and their 2D renderings under various lighting conditions.
• Objects included in this dataset are collected from built environments with artefacts of damage, natural degradation, and high frequency surface textures relevant to engineering disciplines. This includes the presence of cracks and spalling damage which, depending on the fidelity of the applied photometric stereo method, would result in different consequences in civil engineering maintenance applications.
• The materials represented in this dataset are relevant to civil applications (concrete), and further diversified to additional disciplines with inclusion of clay and common plastic used in 3D printing (PLA).
• Additionally, the dataset contains synthetic data with high resolution concrete textures to allow analysis and comparison of experiment virtualization processes with lab collected data.
• Suitable applications for this dataset apply to researchers interested in developing methods for improving the accuracy of photometric stereo and 3D printed objects.
• This data can be used to validate new photometric stereo algorithms by providing photometric stereo input information and their ‘ground truth’ mesh comparisons or developing new methods of generating and validating synthetic data.

## 2 Data Description

**Fig. 1:**
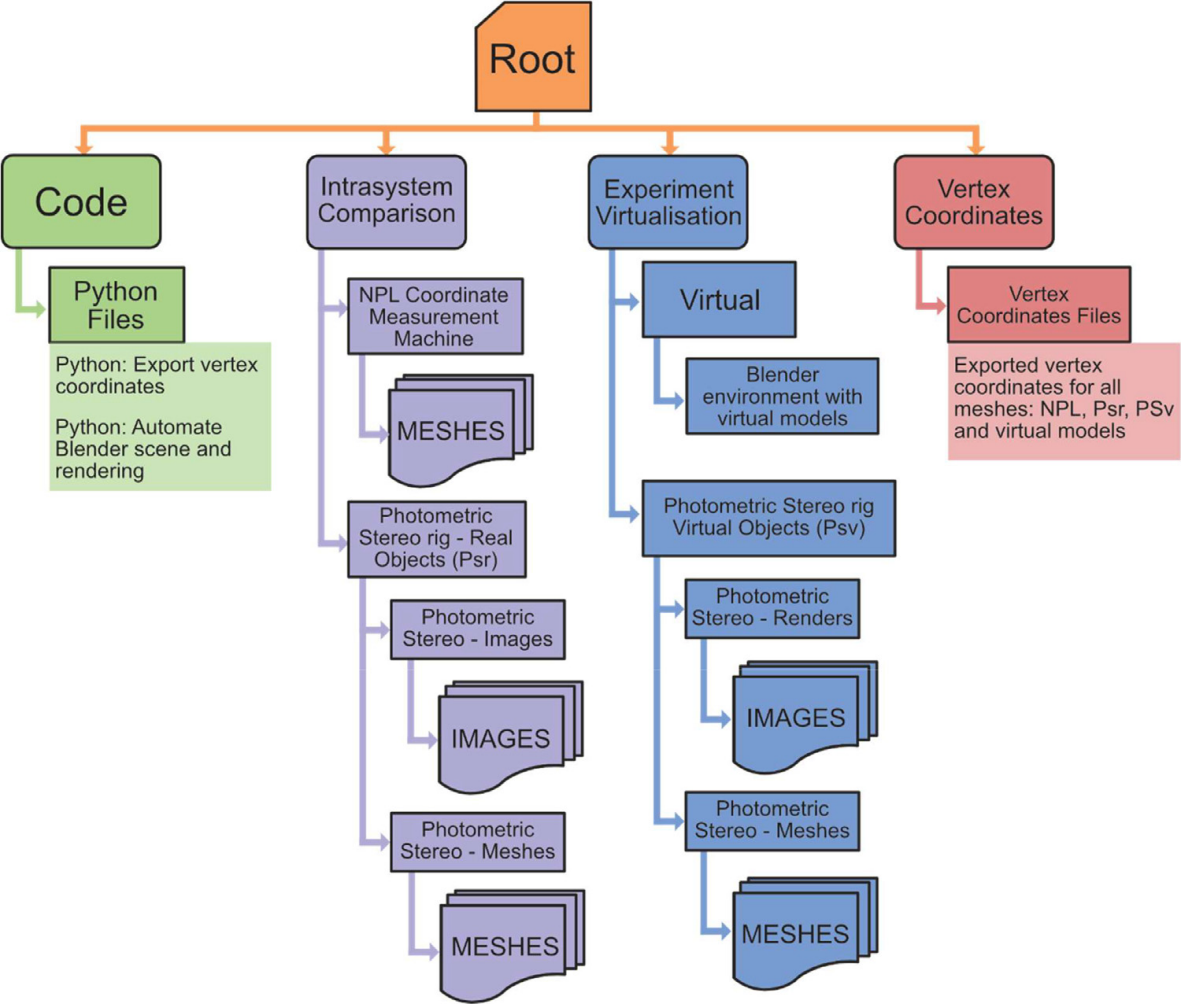
Folder hierarchy, contents, and organisation.

The data is organized into four sections shown in Fig. 1. Supporting code is provided in the ‘Code’ section, with the raw data from both methodology work streams contained in the ‘Intersystem Comparison’ and ‘Experiment Virtualisation’ sections, respectively. The final processed data is contained in the ‘Vertex Coordinates’ section.

### 2.1 Code

This folder contains python code compatible with Blender (Version 3.4). The code files contained are used for executing virtual renders on the virtualized photometric stereo rig in the same manner and naming convention used by the real photometric stereo rig. The other code file is used to extract vertex coordinate data from any mesh to .csv files, to allow the resulting point cloud data to be analysed in different software that are not compatible with 3D object file formats.

- BlenderRender.py
  ○ This python file for Blender scripting automates the rendering process for the virtual rig. This involves ensuring key environmental factors are present and visible in the scene (such as the rig hood, the background, camera, lighting and virtual objects). It sets the visibility of appropriate lighting and objects, executes the render and creates a folder structure to save the output images.
- Blender_ExportAllVertexCoords.py
  ○ A python file for Blender which exports the x, y and z coordinates of every vertex in a selected mesh to a .csv file.

### 2.2 Intersystem comparison

**Fig. 2:**
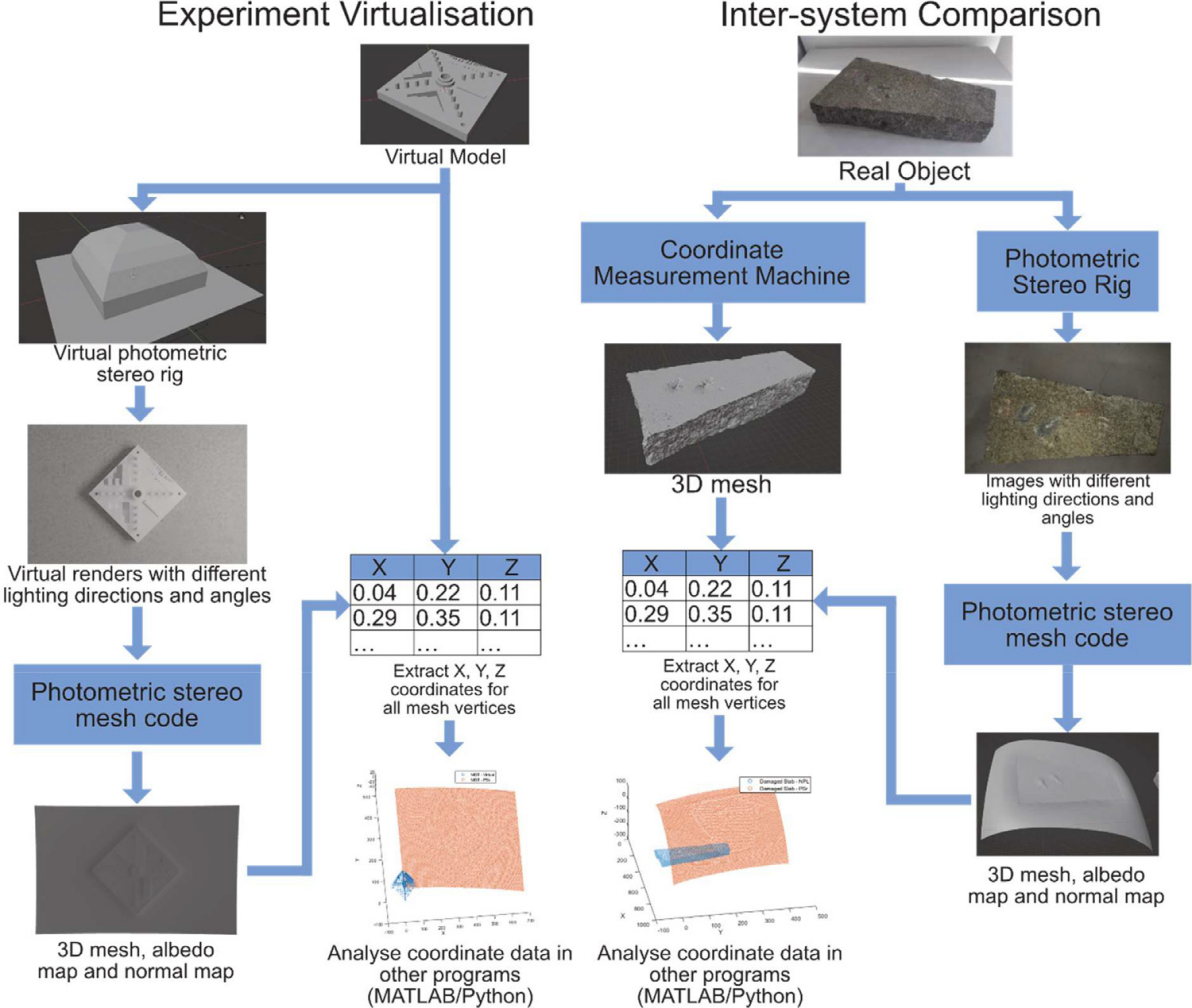
Diagram of intersystem comparison and experiment virtualization workflows.

This folder contains data falling under the intersystem comparison study, the process of which is shown in the right of Fig. 2. The meshes from the coordinate measurement machine at the National Physical Laboratory (NPL), Huddersfield are included, along with the photometric stereo images (pre-photometric stereo code) and the photometric stereo code outputs.

- NPL COORDINATE MEASURMENT MACHINE - REAL
  ○ Coordinate measurement machine device datasheet.
  ○ Text document with experimental metadata, including where objects changed physical location (sphere) and which data was collected by inexperienced users.
  ○ Meshes (stl files) from each of the NPL experiments (Table 1).

**Table 1:** User experience level, number of runs and number of experiments with different object orientations for coordinate measurement machine data.

Object Name	Number of runs	High/Low user experience level	Object orientations
Sphere	7	6 / 1	1
Cylinder	5	1 / 4	1
NIST Additive Manufacturing test artifact	1	1 / 0	1
Rabbit	1	1 / 0	1
Train	1	1 / 0	1
Coral	1	1 / 0	1
Chimney Liner	2	2 / 0	2
Damaged slab	2	2 / 0	2
Broken brick	1	1 / 0	1
- PHOTOMETRIC STEREO – REAL (PSr)
  ○ PSr IMAGES
    ■ Photometric stereo on real objects (input images and rig meta data for each real object. Some objects have multiple orientations).
  ○ PSr MESHES
    ■ Albedo image, normal map, normals used to create the normal map in .npy format and meshes (.ply files). Each experiment has a text file with experimental set up information, such as the height of the support, orientation and number of experiments concerning the same object).

### 2.3 Experiment virtualisation

This folder contains the experiment virtualization study data, which is shown on the left side of Fig. 2. The Blender file containing the virtual rig and virtual objects is available, which includes the camera and render set up, and concrete material assignment to the virtual objects. The python files available in the ‘Code’ folder are used with this file to automate rendering and extract object point cloud data. The renders are available for all virtual objects, and the resulting outputs from the photometric stereo code on these renders.

- VIRTUAL
  ○ Blender file with virtual objects (Figs. 6 and 7), and 8 K concrete materials from [3]
  ○ NIST Additive Manufacturing test artifact file available from [4]
- PHOTOMETRIC STEREO – VIRTUAL (PSv)
  ○ PSv IMAGES
    ■ Photometric stereo on virtual objects (rendered input images and virtual rig meta data)
  ○ PSv MESHES
    ■ Albedo image, normal map, normal used to create the normal map in .npy format and meshes (.ply files).

### 2.4 Vertex coordinates

This folder contains the .csv files of the coordinates of all vertices in all meshes. This covers the NPL coordinate measurement machine determined meshes (NPL prefix), the virtual objects (V prefix) and photometric stereo outputs for the virtual (PSv prefix) and real (PSr prefix) objects. These files can be used in other software for visualization, editing, or further analysis purposes.

## 3 Experimental Design, Materials and Methods

Photometric stereo takes 2D images of an object lit from various angles and the directional information of the lighting sources to recreate the object surface orientation in 3D [5]. Many different applications deploy photometric stereo to faithfully recreate surfaces for further analysis, from heritage digitization in archaeology [6], medical diagnosis in dermatology [7], defect detection in manufacturing [8] and structural health monitoring in civil engineering [9]. However, the underlying process for photometric stereo relies on assumptions on the behaviour of the light sources and surface reflectance which are often violated in practice, making improvements to photometric stereo equipment and algorithms an ongoing process [10].

The data collection methodology in this paper covers two routes detailed in Fig. 2: the intersystem comparison process covers the collection of data for objects under laboratory conditions using a highly characterized coordinate measurement machine and a photometric stereo test rig intended for use in structural health monitoring applications; and the experiment virtualization method develops a virtual, idealised version of the photometric test rig in 3D software to generate renders of virtual objects.

## 4 Intersystem Comparison

**Fig. 3:**
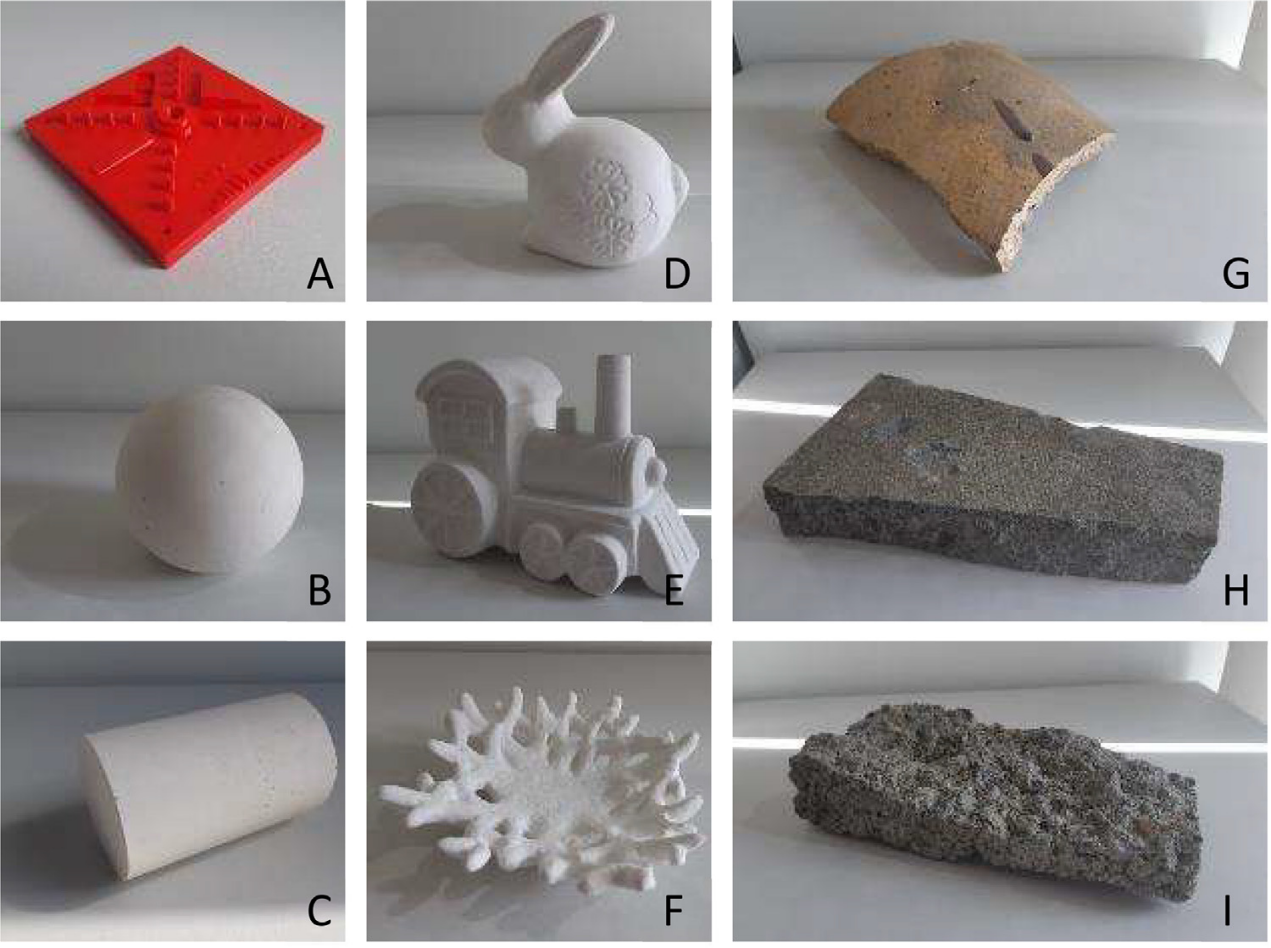
Objects used in the intersystem comparison study, A) 3D printed NIST Additive Manufacturing test artifact (2.85 mm PLA) (approx. 99 × 99 × 17 mm), B) Plaster of Paris sphere (approx. 97 mm diameter), C) Plaster of Paris cylinder (approx. 60 mm diameter, 97 mm length), D) Ceramic rabbit (max dimensions approx. 110 × 67 × 115 mm), E) Ceramic train (max dimensions approx. 150 × 85 × 112 mm), F) Ceramic coral (max dimensions approx. 150 × 145 × 42 mm), G) Chimney liner (unknown material) (max dimensions approx. 200 × 145 × 20 mm), H) Damaged concrete slab ((max dimensions approx. 270 × 144 × 50 mm), I) Broken concrete brick (max dimensions approx. 212 × 94 × 45 mm).

The intersystem comparison covers data collected by the coordinate measurement machine and the photometric stereo test rig. The study covered 9 physical objects with 5 different materials, shown in Fig. 3. The objects were chosen to represent a range of surface features and geometries, such as the plaster cylinder and sphere covering primitives; the plastic 3D printed NIST Additive Manufacturing test artifact [3] acting as a feature reconstruction test; the ceramic household objects such as the rabbit, train and coral, covering intricate, domestic objects; and the concrete damaged slab, chimney segment (unknown material) and broken brick covering structural components of interest to civil applications.

### 4.1 Coordinate measurement machine

**Fig. 4:**
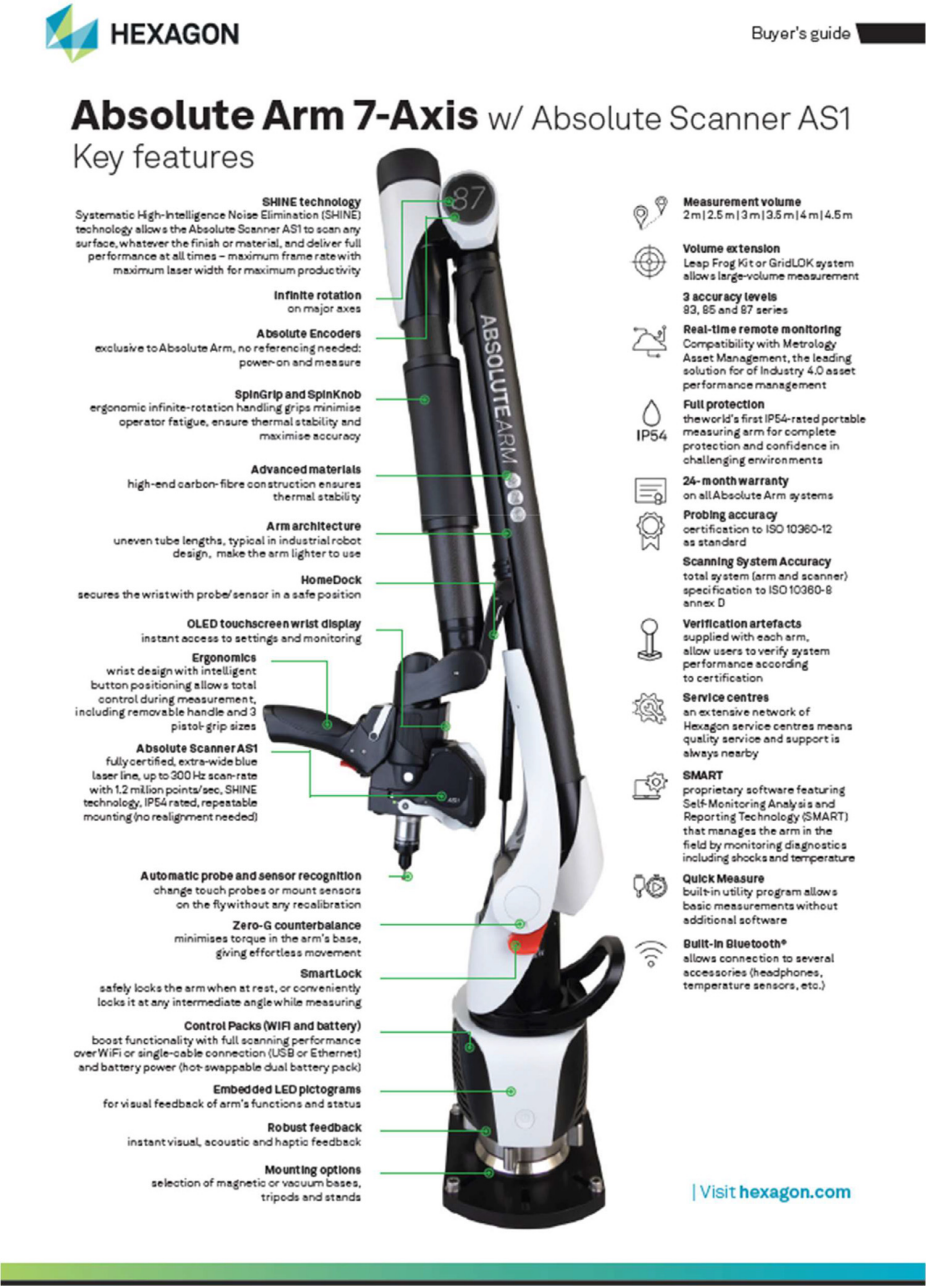
Manufacturer's buyers guide for the model of coordinate measurement machine used in the intersystem comparison.

The coordinate measurement machine used in this study is an articulated arm, model Hexagon Absolute Arm 7-Axis, with a laser scanner end effector, model Hexagon Absolute Scanner AS1, as shown in the manufacturing guide in Fig. 4 [11]. This type of coordinate measurement machine records the location and orientation of the end effector by measuring the rotational position of the joints using precision encoders and subsequently inputs that information into a kinematic model of the arm. Data was collected directly into Polyworks [12] and converted to a polygonal mesh at the point of data collection. The data is exported as an .stl file which can be imported and processed in Blender alongside the output meshes from the photometric stereo test rig.

The experimental set up involved the coordinate measurement machine in range of a support bench where the objects could be placed during the experiments. Experiments were taken multiple times for certain objects to capture the variance between measurements of the same object and were taken by operators with differing levels of experience to capture user error. The information on user experience level, number of runs and number of different orientations for each object is shown in Table 1.

### 4.2 Photometric stereo rig

**Fig. 5:**
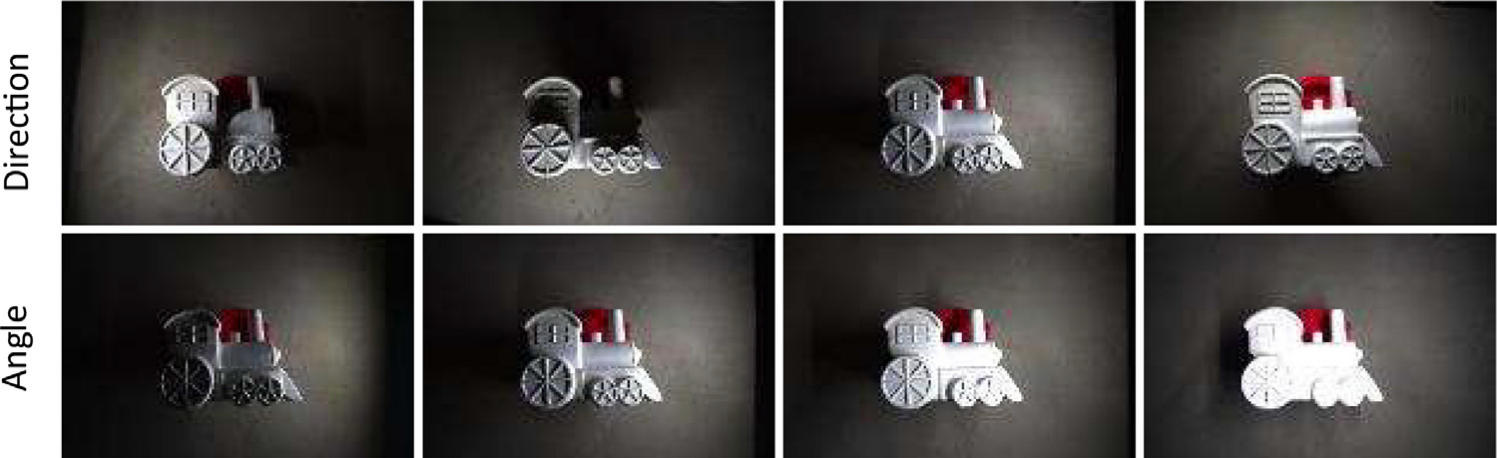
Example output images from the photometric stereo test rig with the train object. The top row shows the different lighting directions (images left to right): lighting from left, bottom, right, top. The bottom row shows the lighting angles (images left to right): object lit from the right by lighting at 10 degrees, 30 degrees, 50 degrees and 70 degrees to the horizontal.

The photometric stereo rig consists of a plastic hood surrounding a camera (Blackfly USB bfs-u3-200s6c, 8 mm lens) with 4 white LED array strips on each of the 4 sides (resulting in 16 LED strips). The positioning of the LED strips allows the object to be illuminated from 4 sides, at 4 angles (10, 30, 50 and 70 degrees to the horizontal). An annotated diagram of the virtual test rig, which was designed to capture the geometry of the real test rig, is shown in Fig. 7. The camera and lighting are trained on the centre of the resting surface of the rig, directly below the camera, with working distances of 250 mm. For each experiment, the camera is calibrated under 70 degree diffuse lighting, where each lighting direction at the same angle of 70 degrees illuminates at once. An image is taken for the object illuminated by each LED strip at each angle, and an image under diffuse lighting is taken for each level, where the 4 LED strips at the same angle light at the same time. This produces 20 images – 4 for each ‘ring’ of LED strips at the same angle to create the diffuse images and 4 different angles on 4 different sides (an image for each of the 16 LED strips). Example images are shown in Fig. 5. These images, along with information concerning the rig and camera set up are given to proprietary photometric stereo software to generate the surface meshes. Further information on the camera and lighting calibration can be found in previous work [13].

The experimental set up for the rig involved elevating the rig on 10 cm high supports at each corner above a flat concrete slab. Objects are placed centrally under the rig, directly below the camera. The rig supports allowed objects to be removed from the rig easily while maintaining alignment. Due to the height of the supports, objects were also placed on supports to allow the area of interest to be within the working distance of the camera. After the objects were placed, a dark shroud made of thick black fabric was placed over the test rig to prevent external light leakage. The test rig can then automate the lighting regime and image collection process to generate the image data. The number of runs per object, different support elevations or object orientations are shown in Table 2.

**Table 2:** Number of runs and number of experiments with different object orientations for photometric stereo data on real objects.

Object Name	Number of runs	Object elevations	Object orientations
Sphere	3	1	1
Cylinder	2	1	2
NIST Additive Manufacturing test artifact	1	1	1
Rabbit	2	1	2
Train	2	1	2
Coral	2	2	1
Chimney Liner	2	1	2
Damaged slab	2	1	2
Broken brick	2	2	1
Blank Background-Benchmark (No objects)	2	2	1
Blank Background-Benchmark (Ruler)	2	2	1

## 5 Experiment Virtualisation

Another method explored to validate the photometric stereo test rig was to develop a virtual version of the set up under ideal conditions. In the real rig, the positions and angles of the components may not directly align with the information provided to the photometric stereo code due to measurement errors. Thus, investigating the impact of variables of interest on the quality of the output mesh may be time consuming, inaccurate and expensive to achieve on the real rig. However, creating lighting at many angles and intensities, or changing the shape and scale of the rig is possible with a virtual rig using precise dimensions. Generating data from virtual objects can remove the influences from inaccuracies in the rig design (such as faulty LEDs or camera misalignment) to test the performance of the photometric stereo code used to convert images to meshes. Additionally, generating data from virtual objects can allow direct comparison with the ground truth for further validation.

### 5.1 Virtual models

**Fig. 6:**
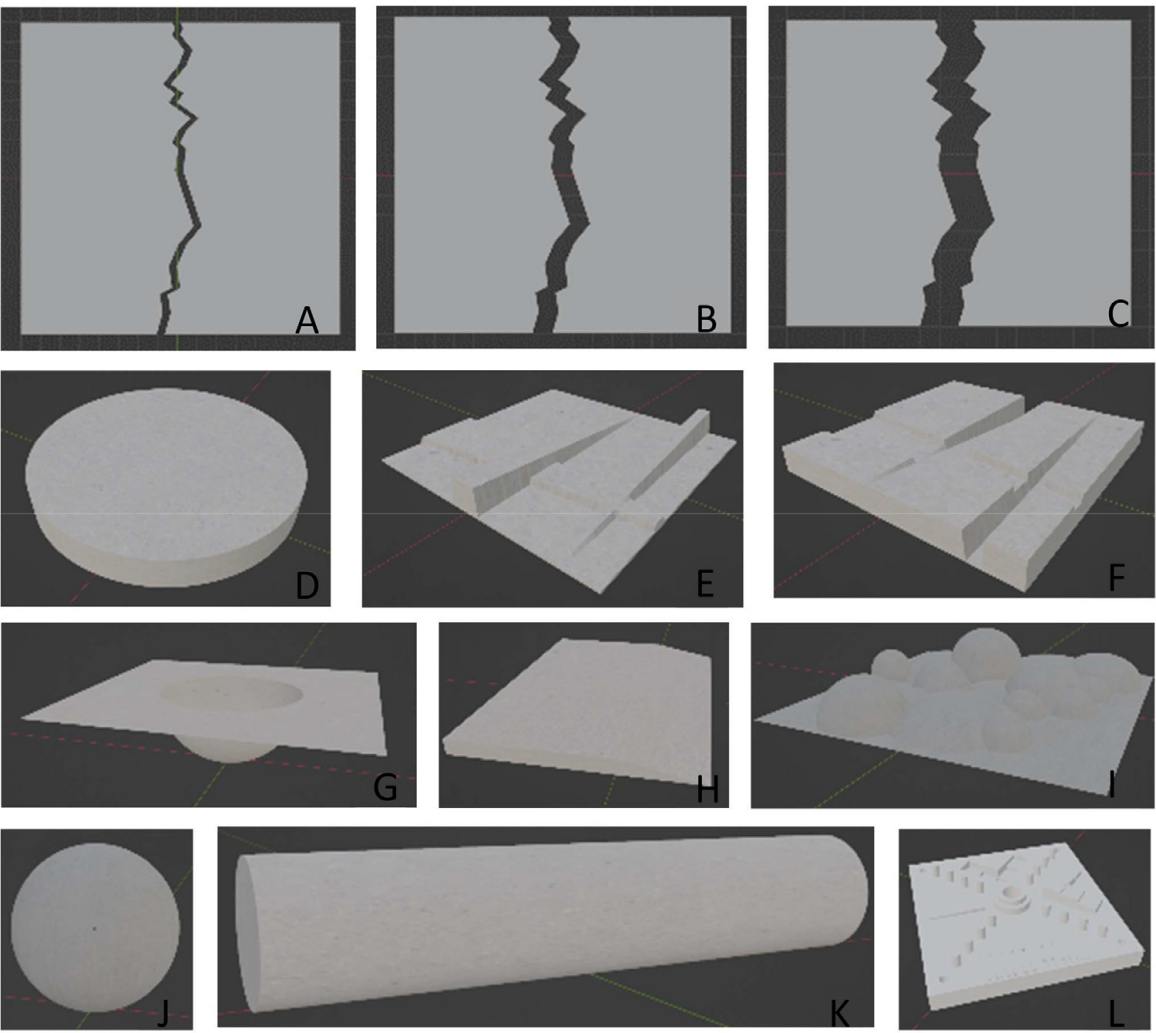
Virtual objects in the experiment virtualization study, A) Cracked slab with 0.5 cm gap, B) Cracked slab with 1 cm gap, C) Cracked slab with 2 cm gap, D) Vertical cylinder, E) Extruded channels with varying width, constant slope and varying slope, constant width, F) Indented channels with varying width, constant slope and varying slope, constant width, G) Plane with hemisphere indent, H) Slab with sloped edge, I) Interlocking spherical textured surface, J) Sphere, K) Cylinder, L) NIST Additive Manufacturing test artifact [4].

Virtual objects created in 3D software act as the ‘ground truth’ for this method, as a perfectly accurate photometric stereo rig would aim to recreate the virtual object exactly. The 3D modelling software of choice is Blender Version 3.4 [2]. To provide more realistic material behaviour, the albedo maps of 8 K resolution mappings of real concrete surfaces were used for the virtual object texture maps [3]. The 3 concrete materials were assigned over 12 virtual models, as shown in Fig. 6. The textures used are included in the provided Blender file. The design of the virtual objects were chosen to investigate the test rig performance on a variety of surface features: 3 objects with a cracked surface of different crack widths to measure the rig capability of capturing damage progression over time; 2 objects with channels of varying width and varying depth (both inset into the surface and extruded out from it) to test the precision limitations of the photometric stereo to gradual changes in surface deformations; a rectangular sloped slab and slab with spherical deformation were created to approximate spalling damage where the surface has been gouged or worn away; a slab with extruded spherical surfaces to understand the limitations of capturing complex surfaces with many shadows cast, not dissimilar to the broken brick object from the real objects; and finally, for consistency with the intersystem comparison, the NIST Additive Manufacturing test artifact [4], sphere and 2 cylinder primitives were created.

### 5.2 Simulation of the photometric stereo rig

**Fig. 7:**
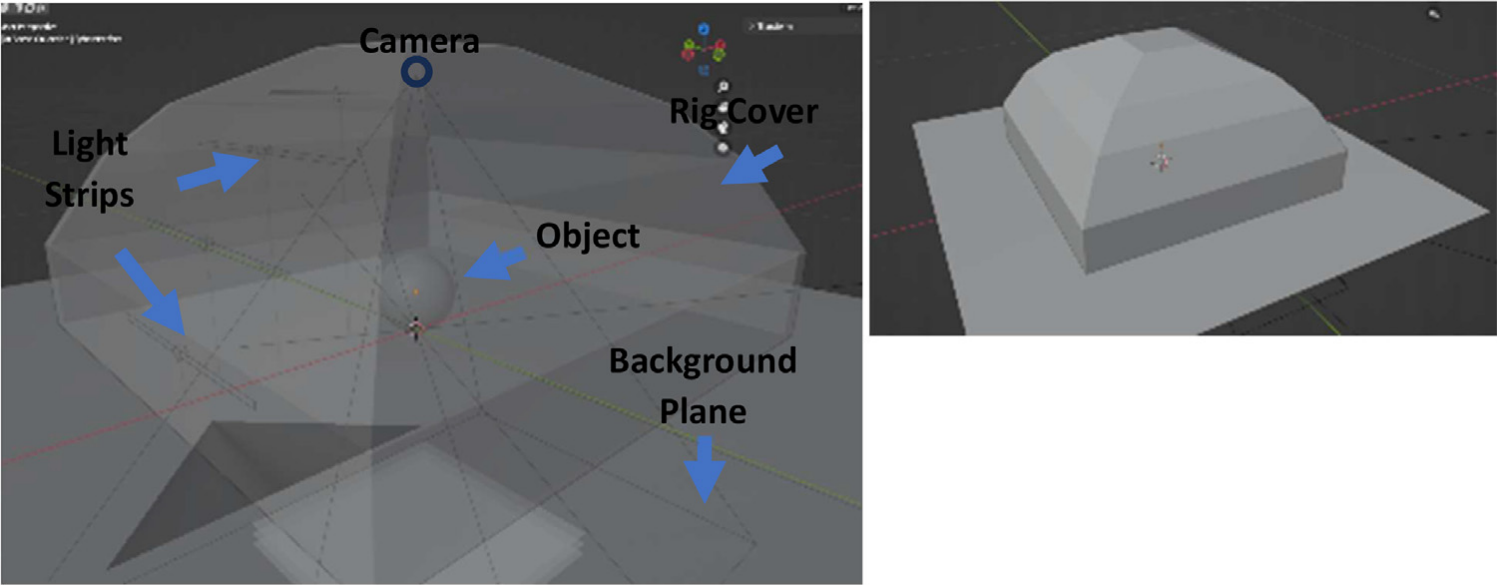
Virtual rig in Blender (Version 3.4), (left) annotated rig shown in semi transparent mode for component visibility, the parts of interest are the background plane, the rig cover, camera, lighting strips and virtual object, (right) shows the rig set up.

Blender (version 3.4) was used to design the test rig, virtual objects and produce rendered images. The dimensions of the virtual rig were created from the information provided to the photometric stereo code. The horizontal and vertical distances of the lights were calculated from the working distance of 250 mm at a given angle (10, 30, 50 or 70 degrees to the horizontal) and the camera parameters were chosen to emulate the test rig camera model datasheet. The render settings were chosen to balance fidelity and simulation runtime to a resolution which matched the test rig output images. The annotated virtual test rig is shown in Fig. 7. The lighting and render regimes were automated through a python script with the image naming scheme and rig meta data produced to match that created by the real rig for compatibility.

### 5.3 Vertex coordinate data

For all methods, the generated 3D mesh is available along with extracted vertex coordinates. To permit the analysis of the meshes in alternative software, all meshes were loaded into Blender (Version 3.4) and a python script was developed to extract the x,y,z coordinates of each vertex in the mesh. This results in a point cloud of data points which represent the mesh. Users of this dataset may also require normal map representations of the output data, which is provided for the Photometric stereo rig outputs. It is possible for this information to be extracted for the coordinate measurement machine and virtual objects through Blender (Version 3.4), as all mesh files generated in this process are compatible with this 3D software.

## Limitations

Limitations due to the photometric stereo test rig include the nature of the camera focus which introduces a depth limitation to capturing sharp images. If the depth of the object exceeds this range, parts of the image may be unfocussed which will impact the output mesh. Additionally, the camera and lights on the test rig are focussed on a certain distance where the objects were elevated to by supports. Any inaccuracies in the height of these supports may impact the camera focus and lighting quality. At the time of writing, the software used to generate the mesh output is not open source. Concerning the experiment virtualisation, Blender does not have high physics fidelity capability which may impact the behaviour of lighting and material interactions. This was counteracted through the use of high resolution scans of real concrete to define the virtual object materials, the object distances could be precisely defined, and the camera was emulated as closely as possible to replicate the real rig under perfect conditions. Additionally, registration between the ground truth and photometric stereo meshes presents an interesting challenge. Other researchers [14] overcome this through the use of features in other 3D software, such as Meshlab's the mutual information method [15]. Finally, the types of applicable photometric stereo algorithms may be affected by the materials in this study, as all real objects possess diffuse, spatially-uniform reflectance. However, these algorithms may still be applicable to the virtual models where the choice of texture can be changed by the user.

## Ethics Statement

This work does not involve human subjects, animal experiments, or any data collected from social media platforms, and the authors have read and adhered to the Data in Brief ethical requirements.

## CRediT authorship contribution statement

**Jennifer Blair:** Conceptualization, Methodology, Software, Investigation, Resources, Data curation, Writing – original draft. **Bruce Stephen:** Conceptualization, Supervision, Writing – review & editing, Project administration, Funding acquisition. **Blair Brown:** Supervision, Writing – review & editing, Project administration, Funding acquisition. **Stephen McArthur:** Writing – review & editing, Project administration, Funding acquisition. **David Gorman:** Investigation, Methodology, Resources. **Alistair Forbes:** Methodology. **Claire Pottier:** Methodology. **Jack McAlorum:** Investigation, Resources, Software. **Hamish Dow:** Investigation, Resources, Software. **Marcus Perry:** Conceptualization, Writing – review & editing.

## Data Availability

Photometric Stereo Data For The Validation Of A Structural Health Monitoring Test Rig (Original data) (PURE).
